# Diaqua­bis­(5-carb­oxy-2-propyl-1*H*-imidazole-4-carboxyl­ato-κ^2^ *N* ^3^,*O* ^4^)zinc(II) 3.5-hydrate

**DOI:** 10.1107/S1600536810031478

**Published:** 2010-08-18

**Authors:** Shi-Jie Li, Wen-Dong Song, Shi-Hong Li, Jing-Jing Dong, Jian-Bin Yan

**Affiliations:** a College of Food Science and Technology, Guang Dong Ocean University, Zhanjiang 524088, People’s Republic of China; b College of Science, Guang Dong Ocean University, Zhanjiang 524088, People’s Republic of China; c College of Medical Laboratory, Hebei North University, Zhangjiakou 075000, People’s Republic of China

## Abstract

In the title complex, [Zn(C_8_H_9_N_2_O_4_)_2_(H_2_O)_2_]·3.5H_2_O, the Zn^II^ ion is coordinated by two *N*,*O*-bidentate H_2_pimda ligands (H_3_pimda = 2-propyl-1*H*-imidazole-4,5-dicarb­oxy­lic acid) and two water mol­ecules in a distorted octa­hedral environment. In the crystal structure, extensive inter­molecular O—H⋯O and N—H⋯O hydrogen bonds stabilize the three-dimensional supra­molecular network. Intra­molecular O—H⋯O hydrogen bonds between the carboxyl groups are also observed. The propyl groups of the two H_2_pimda ligands are disordered each over two sites, with occupancy factors of 0.752 (5):0.248 (5) and 0.519 (7):0.481 (7). One of the water mol­ecules is half-occupied.

## Related literature

For the potential uses and diverse structural types of metal complexes with imidazole-4,5-dicarb­oxy­lic acid, see: Li *et al.* (2006 ▶); Zou *et al.* (2006 ▶). For our previous structural studies of complexes derived from 2-propyl-1*H*-imidazole-4,5-dicarb­oxy­lic acid, see: Fan *et al.* (2010 ▶); He *et al.* (2010 ▶); Li *et al.* (2010 ▶); Song *et al.* (2010 ▶); Yan *et al.* (2010 ▶).
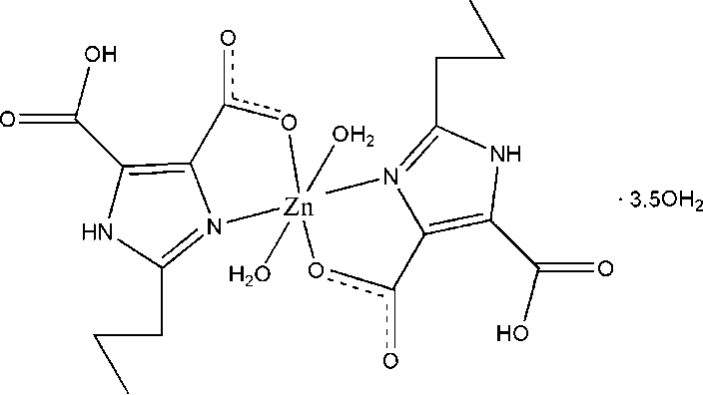


## Experimental

### 

#### Crystal data

[Zn(C_8_H_9_N_2_O_4_)_2_(H_2_O)_2_]·3.5H_2_O
*M* *_r_* = 558.82
Triclinic, 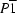

*a* = 10.4780 (18) Å
*b* = 10.5729 (18) Å
*c* = 11.3012 (19) Å
α = 81.783 (2)°
β = 83.035 (2)°
γ = 86.852 (2)°
*V* = 1229.1 (4) Å^3^
*Z* = 2
Mo *K*α radiation
μ = 1.07 mm^−1^
*T* = 296 K
0.29 × 0.24 × 0.21 mm

#### Data collection

Bruker APEXII CCD diffractometer
Absorption correction: multi-scan (*SADABS*; Bruker, 2001 ▶) *T*_min_ = 0.747, *T*_max_ = 0.806
6393 measured reflections
4360 independent reflections
3172 reflections with *I* > 2σ(*I*)
*R*_int_ = 0.033

#### Refinement

*R*[*F* ^2^ > 2σ(*F* ^2^)] = 0.056
*wR*(*F* ^2^) = 0.160
*S* = 1.07
4360 reflections
342 parameters
24 restraints
H-atom parameters constrained
Δρ_max_ = 0.81 e Å^−3^
Δρ_min_ = −0.72 e Å^−3^

### 

Data collection: *APEX2* (Bruker, 2007 ▶); cell refinement: *SAINT* (Bruker, 2007 ▶); data reduction: *SAINT*; program(s) used to solve structure: *SHELXS97* (Sheldrick, 2008 ▶); program(s) used to refine structure: *SHELXL97* (Sheldrick, 2008 ▶); molecular graphics: *SHELXTL* (Sheldrick, 2008 ▶); software used to prepare material for publication: *SHELXTL*.

## Supplementary Material

Crystal structure: contains datablocks I, 1R. DOI: 10.1107/S1600536810031478/hy2336sup1.cif

Structure factors: contains datablocks I. DOI: 10.1107/S1600536810031478/hy2336Isup2.hkl

Additional supplementary materials:  crystallographic information; 3D view; checkCIF report

## Figures and Tables

**Table 1:** Hydrogen-bond geometry (Å, °)

*D*—H⋯*A*	*D*—H	H⋯*A*	*D*⋯*A*	*D*—H⋯*A*
O1—H1⋯O4	0.82	1.68	2.502 (6)	180
O7—H7⋯O6	0.82	1.63	2.445 (6)	176
N2—H2⋯O4*W*	0.86	1.88	2.737 (6)	171
N4—H4⋯O6*W*	0.86	2.03	2.863 (6)	162
O1*W*—H1*W*⋯O5*W*^i^	0.84	1.86	2.675 (6)	164
O1*W*—H2*W*⋯O8^ii^	0.84	1.89	2.720 (5)	170
O2*W*—H3*W*⋯O8^iii^	0.83	2.13	2.910 (6)	155
O2*W*—H4*W*⋯O2^iv^	0.83	2.00	2.802 (6)	161
O3*W*—H5*W*⋯O3^iv^	0.84	1.98	2.795 (8)	162
O3*W*—H6*W*⋯O3^v^	0.84	1.92	2.743 (8)	165
O4*W*—H7*W*⋯O3*W*	0.84	1.81	2.633 (9)	163
O4*W*—H8*W*⋯O7^vi^	0.84	2.16	2.877 (6)	143
O5*W*—H9*W*⋯O5^vi^	0.85	2.14	2.865 (6)	144
O5*W*—H10*W*⋯O4^vii^	0.85	1.98	2.812 (6)	169
O6*W*—H11*W*⋯O3*W*^vii^	0.84	1.74	2.574 (10)	174
O6*W*—H12*W*⋯O5*W*^viii^	0.84	2.27	2.789 (7)	120

Symmetry codes: (i) 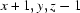; (ii) 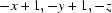; (iii) 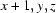; (iv) 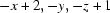; (v) 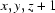; (vi) 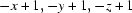; (vii) 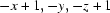; (viii) 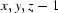.

**Fig. 1.**
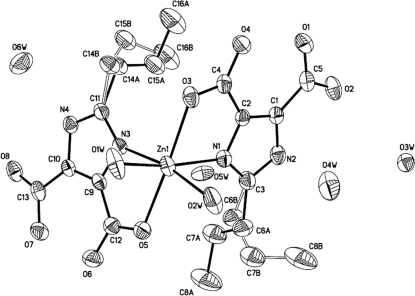
Molecular structure of the title compound. Displacement ellipsoids are shown at the 30% probability level. Open bonds show minor disordered sites. H atoms have been omitted for clarity.

## supplementary crystallographic information

### Comment

Recently, our research group has shown great interest in the solid-state coordination chemistry of N-heterocyclic carboxylic acids, such as 2-propyl-1*H*-imidazole-4,5-dicarboxylic acid (H_3_pimda) ligand as a derivative of imidazole-4,5-dicarboxylic acid (H_3_idc). The efficient N,O-donors have been used to obtain new metal-organic complexes by our research group, such as poly[diaquabis(5-carboxy-2-propyl-1*H*-imidazole-4-carboxylato-κ^3^ N^3^,O^4^,O^5^)calcium(II)] (Song *et al.*, 2010), [diaquabis(5-carboxy-2-propyl-1*H*-imidazole-4-carboxylato- κ^2^N^3^,O^4^)manganese(II)] N,N-dimethylformamide (Yan *et al.*, 2010), [diaquabis(5-carboxy-2-propyl-1*H*-imidazole-4-carboxylato- κ^2^N^3^,O^4^)nickle(II)] N,N-dimethylformamide disolvate (Li *et al.*, 2010), diaquabis(4-carboxy-2-propyl-1*H*-imidazole-5-carboxylato- κ^2^N^3^,O^4^)copper(II) N,N-dimethylformamide disolvate (He *et al.*, 2010) and diaquabis(5-carboxy-2-propyl-1*H*-imidazole-4-carboxylato- κ^2^N^3^,O^4^)nickle(II) tetrahedrate (Fan *et al.*, 2010). In this paper, we report the structure of a new zinc(II) complex with H_2_pimda obtained under hydrothermal conditions.

As illustrated in Fig. 1, the title complex is isomorphous with its Ni(II) analogue (Fan *et al.*, 2010). Similar structural descriptions can be applied to the present isomorphous complex. The Zn^II^ atom is six-coordinated by two N,O-bidentate H_2_pimda ligands and two water molecules in a distorted octahedral geometry. The dihedral angle between the two imidazole rings is 77.8 (5)°. In the crystal structure, the three-dimensional supramolecular network is stabilized by extensive O—H···O and N—H···O hydrogen bonds involving the uncoordinated and coordinated water molecules, the carboxy groups and the protonated N atoms of the imidazole rings (Table 1). The propyl groups of the H_2_pimda ligands are disordered each over two sites, with refined occupancies of 0.752 (5): 0.248 (5) and 0.519 (7):0.481 (7).

### Experimental

A mixture of Zn(NO_3_)_2_ (0.5 mmol, 0.09 g) and 2-propyl-1*H*-imidazole-4,5-dicarboxylic acid (0.5 mmol, 0.99 g) in 15 ml of H_2_O solution was sealed in an autoclave equipped with a Teflon liner (20 ml) and then heated at 433 K for 4 d. Crystals of the title compound were obtained by slow evaporation of the solvent at room temperature.

### Refinement

C- and N-bound H atoms were placed at calculated positions and were treated as riding on the parent atoms, with C—H = 0.97 (CH_2_) and 0.96 (CH_3_) Å, N—H = 0.86 Å and with *U*_iso_(H) = 1.2(1.5 for methyl)*U*_eq_(C, N). H atoms of the water molecules were located in a difference Fourier map and were allowed to ride on the parent atom, with *U*_iso_(H) = 1.5*U*_eq_(O). The propyl groups of the H_2_pimda ligands are splited into two sets of sites, with refined occupancies of 0.752 (5):0.248 (5) and 0.519 (7):0.481 (7). One of the water molecules is half-occupied.

### Crystal data

**Table:**

[Zn(C_8_H_9_N_2_O_4_)_2_(H_2_O)_2_]·3.5H_2_O	*Z* = 2
*M**_r_* = 558.82	*F*(000) = 582
Triclinic, *P*1	*D*_x_ = 1.510 Mg m^−^^3^
Hall symbol: -P 1	Mo *K*α radiation, λ = 0.71073 Å
*a* = 10.4780 (18) Å	Cell parameters from 3600 reflections
*b* = 10.5729 (18) Å	θ = 1.4–28°
*c* = 11.3012 (19) Å	µ = 1.07 mm^−^^1^
α = 81.783 (2)°	*T* = 296 K
β = 83.035 (2)°	Block, colorless
γ = 86.852 (2)°	0.29 × 0.24 × 0.21 mm
*V* = 1229.1 (4) Å^3^	

### Data collection

**Table:**

Bruker APEXII CCD diffractometer	4360 independent reflections
Radiation source: fine-focus sealed tube	3172 reflections with *I* > 2σ(*I*)
graphite	*R*_int_ = 0.033
φ and ω scan	θ_max_ = 25.2°, θ_min_ = 1.8°
Absorption correction: multi-scan (*SADABS*; Bruker, 2001)	*h* = −10→12
*T*_min_ = 0.747, *T*_max_ = 0.806	*k* = −11→12
6393 measured reflections	*l* = −11→13

### Refinement

**Table:**

Refinement on *F*^2^	Primary atom site location: structure-invariant direct methods
Least-squares matrix: full	Secondary atom site location: difference Fourier map
*R*[*F*^2^ > 2σ(*F*^2^)] = 0.056	Hydrogen site location: inferred from neighbouring sites
*wR*(*F*^2^) = 0.160	H-atom parameters constrained
*S* = 1.07	*w* = 1/[σ^2^(*F*_o_^2^) + (0.0755*P*)^2^ + 0.5831*P*] where *P* = (*F*_o_^2^ + 2*F*_c_^2^)/3
4360 reflections	(Δ/σ)_max_ < 0.001
342 parameters	Δρ_max_ = 0.81 e Å^−^^3^
24 restraints	Δρ_min_ = −0.72 e Å^−^^3^

### Fractional atomic coordinates and isotropic or equivalent isotropic displacement parameters (Å^2^)

**Table:**

	*x*	*y*	*z*	*U*_iso_*/*U*_eq_	Occ. (<1)
Zn1	0.84596 (6)	0.28957 (6)	0.19279 (5)	0.0468 (3)	
O1	0.8745 (5)	−0.1862 (4)	0.5169 (4)	0.0652 (12)	
H1	0.8821	−0.1583	0.4450	0.098*	
O2	0.8364 (4)	−0.1087 (4)	0.6884 (4)	0.0677 (12)	
O3	0.8898 (4)	0.0899 (4)	0.1842 (3)	0.0505 (9)	
O4	0.8972 (4)	−0.1006 (4)	0.2975 (3)	0.0604 (11)	
O5	0.7797 (4)	0.4859 (3)	0.2071 (3)	0.0522 (10)	
O6	0.6119 (4)	0.6225 (4)	0.1874 (4)	0.0647 (11)	
O7	0.3908 (4)	0.6171 (4)	0.1402 (4)	0.0595 (11)	
H7	0.4644	0.6156	0.1581	0.089*	
O8	0.2581 (4)	0.4768 (4)	0.0987 (4)	0.0590 (11)	
N1	0.8293 (4)	0.2124 (4)	0.3764 (4)	0.0432 (10)	
N2	0.8107 (4)	0.1425 (4)	0.5690 (4)	0.0521 (12)	
H2	0.7979	0.1424	0.6456	0.062*	
N3	0.6451 (4)	0.2860 (4)	0.1716 (4)	0.0399 (10)	
N4	0.4524 (4)	0.2803 (4)	0.1319 (4)	0.0466 (11)	
H4	0.3826	0.2499	0.1168	0.056*	
C1	0.8351 (5)	0.0371 (5)	0.5118 (4)	0.0412 (12)	
C2	0.8473 (4)	0.0830 (5)	0.3922 (4)	0.0386 (11)	
C3	0.8102 (6)	0.2472 (6)	0.4847 (5)	0.0561 (15)	
C4	0.8810 (5)	0.0187 (5)	0.2833 (5)	0.0444 (13)	
C5	0.8477 (5)	−0.0920 (6)	0.5795 (5)	0.0517 (14)	
C6A	0.8065 (10)	0.3828 (11)	0.5113 (11)	0.070 (3)	0.753 (7)
H6A	0.8542	0.4358	0.4453	0.083*	0.753 (7)
H6B	0.8468	0.3860	0.5836	0.083*	0.753 (7)
C7A	0.6671 (10)	0.4347 (9)	0.5286 (9)	0.083 (3)	0.753 (7)
H7A	0.6233	0.4217	0.4607	0.100*	0.753 (7)
H7B	0.6222	0.3891	0.6010	0.100*	0.753 (7)
C8A	0.6658 (14)	0.5762 (10)	0.5390 (12)	0.129 (5)	0.753 (7)
H8A	0.5796	0.6054	0.5640	0.193*	0.753 (7)
H8B	0.6957	0.6228	0.4623	0.193*	0.753 (7)
H8C	0.7211	0.5900	0.5973	0.193*	0.753 (7)
C6B	0.742 (4)	0.367 (4)	0.522 (4)	0.070 (3)	0.247 (7)
H6C	0.6885	0.3479	0.5983	0.083*	0.247 (7)
H6D	0.6894	0.4072	0.4618	0.083*	0.247 (7)
C7B	0.852 (3)	0.453 (3)	0.535 (3)	0.083 (3)	0.247 (7)
H7C	0.9083	0.4631	0.4596	0.100*	0.247 (7)
H7D	0.8155	0.5368	0.5476	0.100*	0.247 (7)
C8B	0.933 (4)	0.405 (3)	0.636 (3)	0.129 (5)	0.247 (7)
H8D	0.9985	0.4647	0.6367	0.193*	0.247 (7)
H8E	0.9724	0.3233	0.6228	0.193*	0.247 (7)
H8F	0.8792	0.3969	0.7111	0.193*	0.247 (7)
C9	0.5881 (5)	0.4058 (5)	0.1698 (4)	0.0401 (12)	
C10	0.4645 (5)	0.4030 (5)	0.1425 (4)	0.0408 (12)	
C11	0.5583 (5)	0.2111 (5)	0.1471 (5)	0.0464 (13)	
C12	0.6653 (5)	0.5103 (5)	0.1892 (5)	0.0470 (13)	
C13	0.3619 (5)	0.5037 (6)	0.1269 (5)	0.0476 (14)	
C14A	0.560 (3)	0.0677 (8)	0.1631 (19)	0.057 (4)	0.525 (10)
H14A	0.6447	0.0341	0.1364	0.069*	0.525 (10)
H14B	0.4986	0.0391	0.1157	0.069*	0.525 (10)
C15A	0.522 (2)	0.0179 (12)	0.3023 (15)	0.079 (4)	0.525 (10)
H15A	0.5775	0.0541	0.3504	0.095*	0.525 (10)
H15B	0.4337	0.0440	0.3269	0.095*	0.525 (10)
C16A	0.5373 (18)	−0.1247 (14)	0.3208 (18)	0.114 (6)	0.525 (10)
H16A	0.6111	−0.1500	0.3625	0.171*	0.525 (10)
H16B	0.5487	−0.1554	0.2441	0.171*	0.525 (10)
H16C	0.4618	−0.1602	0.3677	0.171*	0.525 (10)
C14B	0.590 (3)	0.0766 (10)	0.122 (2)	0.057 (4)	0.475 (10)
H14C	0.6787	0.0534	0.1332	0.069*	0.475 (10)
H14D	0.5784	0.0700	0.0387	0.069*	0.475 (10)
C15B	0.4966 (14)	−0.0173 (14)	0.2106 (15)	0.079 (4)	0.475 (10)
H15C	0.4078	0.0101	0.2028	0.095*	0.475 (10)
H15D	0.5095	−0.1032	0.1895	0.095*	0.475 (10)
C16B	0.523 (3)	−0.017 (2)	0.3369 (18)	0.114 (6)	0.475 (10)
H16D	0.4858	−0.0906	0.3866	0.171*	0.475 (10)
H16E	0.4861	0.0592	0.3656	0.171*	0.475 (10)
H16F	0.6143	−0.0210	0.3401	0.171*	0.475 (10)
O1W	0.8877 (4)	0.3308 (5)	0.0105 (4)	0.0795 (15)	
H1W	0.9491	0.2983	−0.0309	0.119*	
H2W	0.8511	0.3925	−0.0291	0.119*	
O2W	1.0370 (4)	0.3313 (4)	0.2110 (4)	0.0789 (14)	
H3W	1.0823	0.3900	0.1747	0.118*	
H4W	1.0782	0.2755	0.2525	0.118*	
O3W	0.9078 (8)	0.0156 (7)	0.9598 (7)	0.058 (2)	0.50
H5W	0.9780	−0.0146	0.9293	0.087*	0.50
H6W	0.9159	0.0441	1.0240	0.087*	0.50
O4W	0.7783 (6)	0.1691 (6)	0.8082 (4)	0.123 (2)	
H7W	0.8323	0.1232	0.8463	0.185*	
H8W	0.7604	0.2379	0.8361	0.185*	
O5W	0.1009 (4)	0.2742 (4)	0.8694 (4)	0.0798 (14)	
H9W	0.1112	0.3450	0.8245	0.120*	
H10W	0.0969	0.2146	0.8273	0.120*	
O6W	0.2574 (5)	0.1562 (6)	0.0392 (5)	0.111 (2)	
H11W	0.2034	0.0991	0.0448	0.167*	
H12W	0.2230	0.2297	0.0243	0.167*	

### Atomic displacement parameters (Å^2^)

**Table:**

	*U*^11^	*U*^22^	*U*^33^	*U*^12^	*U*^13^	*U*^23^
Zn1	0.0433 (4)	0.0509 (4)	0.0425 (4)	0.0042 (3)	−0.0065 (3)	0.0058 (3)
O1	0.080 (3)	0.051 (2)	0.060 (3)	0.000 (2)	−0.014 (2)	0.009 (2)
O2	0.067 (3)	0.081 (3)	0.044 (2)	0.009 (2)	−0.002 (2)	0.017 (2)
O3	0.056 (2)	0.058 (2)	0.034 (2)	0.0147 (18)	−0.0051 (16)	−0.0036 (17)
O4	0.083 (3)	0.049 (2)	0.050 (2)	0.011 (2)	−0.016 (2)	−0.0082 (19)
O5	0.049 (2)	0.049 (2)	0.060 (2)	−0.0048 (18)	−0.0141 (18)	−0.0019 (18)
O6	0.071 (3)	0.044 (2)	0.081 (3)	0.003 (2)	−0.016 (2)	−0.014 (2)
O7	0.049 (2)	0.054 (2)	0.074 (3)	0.0143 (19)	−0.010 (2)	−0.006 (2)
O8	0.040 (2)	0.065 (3)	0.066 (3)	0.0044 (19)	−0.0078 (19)	0.011 (2)
N1	0.044 (3)	0.046 (3)	0.038 (2)	0.002 (2)	−0.0067 (19)	−0.004 (2)
N2	0.057 (3)	0.063 (3)	0.035 (2)	0.007 (2)	−0.005 (2)	−0.006 (2)
N3	0.038 (2)	0.037 (2)	0.043 (2)	0.0037 (19)	−0.0076 (18)	0.0008 (18)
N4	0.040 (3)	0.049 (3)	0.049 (3)	−0.006 (2)	−0.008 (2)	0.003 (2)
C1	0.035 (3)	0.048 (3)	0.039 (3)	−0.003 (2)	−0.003 (2)	0.000 (2)
C2	0.033 (3)	0.043 (3)	0.039 (3)	−0.003 (2)	−0.008 (2)	−0.002 (2)
C3	0.071 (4)	0.052 (3)	0.046 (3)	0.009 (3)	−0.013 (3)	−0.007 (3)
C4	0.040 (3)	0.052 (3)	0.041 (3)	0.007 (2)	−0.011 (2)	−0.007 (3)
C5	0.041 (3)	0.062 (4)	0.048 (3)	0.001 (3)	−0.007 (3)	0.007 (3)
C6A	0.091 (10)	0.058 (6)	0.062 (5)	−0.003 (7)	−0.010 (7)	−0.015 (4)
C7A	0.100 (8)	0.062 (6)	0.086 (7)	0.005 (6)	0.000 (6)	−0.015 (5)
C8A	0.182 (14)	0.063 (6)	0.136 (11)	0.022 (7)	0.006 (10)	−0.023 (7)
C6B	0.091 (10)	0.058 (6)	0.062 (5)	−0.003 (7)	−0.010 (7)	−0.015 (4)
C7B	0.100 (8)	0.062 (6)	0.086 (7)	0.005 (6)	0.000 (6)	−0.015 (5)
C8B	0.182 (14)	0.063 (6)	0.136 (11)	0.022 (7)	0.006 (10)	−0.023 (7)
C9	0.043 (3)	0.039 (3)	0.034 (3)	0.002 (2)	−0.001 (2)	0.004 (2)
C10	0.042 (3)	0.040 (3)	0.037 (3)	−0.001 (2)	−0.005 (2)	0.004 (2)
C11	0.045 (3)	0.042 (3)	0.052 (3)	0.001 (3)	−0.014 (2)	0.001 (2)
C12	0.048 (3)	0.047 (3)	0.045 (3)	−0.002 (3)	−0.003 (2)	−0.005 (2)
C13	0.042 (3)	0.057 (4)	0.038 (3)	0.002 (3)	0.003 (2)	0.006 (3)
C14A	0.035 (12)	0.042 (4)	0.092 (13)	−0.007 (4)	−0.007 (9)	0.003 (5)
C15A	0.066 (7)	0.050 (6)	0.121 (11)	−0.004 (5)	−0.029 (7)	0.005 (7)
C16A	0.111 (11)	0.078 (9)	0.139 (13)	0.004 (10)	−0.010 (9)	0.023 (10)
C14B	0.035 (12)	0.042 (4)	0.092 (13)	−0.007 (4)	−0.007 (9)	0.003 (5)
C15B	0.066 (7)	0.050 (6)	0.121 (11)	−0.004 (5)	−0.029 (7)	0.005 (7)
C16B	0.111 (11)	0.078 (9)	0.139 (13)	0.004 (10)	−0.010 (9)	0.023 (10)
O1W	0.065 (3)	0.106 (4)	0.049 (2)	0.039 (3)	0.007 (2)	0.025 (2)
O2W	0.049 (3)	0.085 (3)	0.091 (3)	−0.013 (2)	−0.019 (2)	0.042 (3)
O3W	0.066 (5)	0.065 (5)	0.045 (4)	0.005 (4)	−0.007 (4)	−0.017 (4)
O4W	0.166 (6)	0.143 (5)	0.067 (3)	0.070 (5)	−0.032 (4)	−0.053 (3)
O5W	0.077 (3)	0.074 (3)	0.090 (3)	−0.022 (2)	0.016 (3)	−0.031 (3)
O6W	0.109 (5)	0.104 (4)	0.140 (5)	0.005 (3)	−0.061 (4)	−0.048 (4)

### Geometric parameters (Å, °)

**Table:**

Zn1—O1W	2.042 (4)	C6B—H6D	0.9700
Zn1—N1	2.108 (4)	C7B—C8B	1.512 (17)
Zn1—O2W	2.113 (4)	C7B—H7C	0.9700
Zn1—O3	2.149 (4)	C7B—H7D	0.9700
Zn1—N3	2.149 (4)	C8B—H8D	0.9600
Zn1—O5	2.174 (4)	C8B—H8E	0.9600
O1—C5	1.301 (7)	C8B—H8F	0.9600
O1—H1	0.8200	C9—C10	1.370 (7)
O2—C5	1.210 (6)	C9—C12	1.460 (8)
O3—C4	1.253 (6)	C10—C13	1.479 (7)
O4—C4	1.254 (6)	C11—C14A	1.500 (10)
O5—C12	1.246 (6)	C11—C14B	1.502 (11)
O6—C12	1.282 (6)	C14A—C15A	1.594 (18)
O7—C13	1.286 (7)	C14A—H14A	0.9700
O7—H7	0.8200	C14A—H14B	0.9700
O8—C13	1.229 (7)	C15A—C16A	1.494 (15)
N1—C3	1.318 (7)	C15A—H15A	0.9700
N1—C2	1.360 (6)	C15A—H15B	0.9700
N2—C3	1.353 (7)	C16A—H16A	0.9600
N2—C1	1.363 (7)	C16A—H16B	0.9600
N2—H2	0.8600	C16A—H16C	0.9600
N3—C11	1.318 (7)	C14B—C15B	1.592 (18)
N3—C9	1.369 (6)	C14B—H14C	0.9700
N4—C11	1.311 (6)	C14B—H14D	0.9700
N4—C10	1.334 (7)	C15B—C16B	1.487 (17)
N4—H4	0.8600	C15B—H15C	0.9700
C1—C2	1.363 (7)	C15B—H15D	0.9700
C1—C5	1.474 (7)	C16B—H16D	0.9600
C2—C4	1.486 (7)	C16B—H16E	0.9600
C3—C6A	1.504 (13)	C16B—H16F	0.9600
C3—C6B	1.51 (4)	O1W—H1W	0.8363
C6A—C7A	1.531 (13)	O1W—H2W	0.8393
C6A—H6A	0.9700	O2W—H3W	0.8323
C6A—H6B	0.9700	O2W—H4W	0.8337
C7A—C8A	1.517 (13)	O3W—H5W	0.8402
C7A—H7A	0.9700	O3W—H6W	0.8405
C7A—H7B	0.9700	O4W—H7W	0.8420
C8A—H8A	0.9600	O4W—H8W	0.8374
C8A—H8B	0.9600	O5W—H9W	0.8466
C8A—H8C	0.9600	O5W—H10W	0.8485
C6B—C7B	1.539 (17)	O6W—H11W	0.8409
C6B—H6C	0.9700	O6W—H12W	0.8416
			
O1W—Zn1—N1	167.71 (16)	C6B—C7B—H7C	108.4
O1W—Zn1—O2W	88.74 (19)	C8B—C7B—H7D	108.4
N1—Zn1—O2W	87.04 (16)	C6B—C7B—H7D	108.4
O1W—Zn1—O3	90.84 (16)	H7C—C7B—H7D	107.5
N1—Zn1—O3	77.98 (14)	C7B—C8B—H8D	109.5
O2W—Zn1—O3	94.19 (17)	C7B—C8B—H8E	109.5
O1W—Zn1—N3	89.81 (17)	H8D—C8B—H8E	109.5
N1—Zn1—N3	96.47 (16)	C7B—C8B—H8F	109.5
O2W—Zn1—N3	169.05 (16)	H8D—C8B—H8F	109.5
O3—Zn1—N3	96.68 (15)	H8E—C8B—H8F	109.5
O1W—Zn1—O5	91.52 (16)	N3—C9—C10	109.4 (5)
N1—Zn1—O5	100.14 (15)	N3—C9—C12	118.4 (5)
O2W—Zn1—O5	91.72 (16)	C10—C9—C12	132.1 (5)
O3—Zn1—O5	173.69 (14)	N4—C10—C9	103.1 (4)
N3—Zn1—O5	77.47 (15)	N4—C10—C13	124.6 (5)
C5—O1—H1	109.5	C9—C10—C13	132.3 (5)
C4—O3—Zn1	115.7 (3)	N4—C11—N3	108.1 (5)
C12—O5—Zn1	114.6 (3)	N4—C11—C14A	121.7 (13)
C13—O7—H7	109.5	N3—C11—C14A	128.5 (14)
C3—N1—C2	106.5 (4)	N4—C11—C14B	128.3 (15)
C3—N1—Zn1	141.4 (4)	N3—C11—C14B	122.8 (15)
C2—N1—Zn1	112.0 (3)	O5—C12—O6	123.2 (5)
C3—N2—C1	108.5 (4)	O5—C12—C9	118.4 (5)
C3—N2—H2	125.8	O6—C12—C9	118.3 (5)
C1—N2—H2	125.8	O8—C13—O7	124.2 (5)
C11—N3—C9	106.7 (4)	O8—C13—C10	119.6 (5)
C11—N3—Zn1	142.0 (3)	O7—C13—C10	116.2 (5)
C9—N3—Zn1	110.9 (3)	C11—C14A—C15A	108.3 (10)
C11—N4—C10	112.7 (5)	C11—C14A—H14A	110.0
C11—N4—H4	123.6	C15A—C14A—H14A	110.0
C10—N4—H4	123.6	C11—C14A—H14B	110.0
N2—C1—C2	105.1 (4)	C15A—C14A—H14B	110.0
N2—C1—C5	121.5 (5)	H14A—C14A—H14B	108.4
C2—C1—C5	133.4 (5)	C16A—C15A—C14A	108.5 (13)
N1—C2—C1	110.1 (4)	C16A—C15A—H15A	110.0
N1—C2—C4	117.9 (4)	C14A—C15A—H15A	110.0
C1—C2—C4	131.9 (5)	C16A—C15A—H15B	110.0
N1—C3—N2	109.8 (5)	C14A—C15A—H15B	110.0
N1—C3—C6A	125.1 (7)	H15A—C15A—H15B	108.4
N2—C3—C6A	124.6 (7)	C11—C14B—C15B	108.7 (12)
N1—C3—C6B	128.2 (18)	C11—C14B—H14C	109.9
N2—C3—C6B	117.2 (18)	C15B—C14B—H14C	109.9
O3—C4—O4	125.6 (5)	C11—C14B—H14D	109.9
O3—C4—C2	116.3 (5)	C15B—C14B—H14D	109.9
O4—C4—C2	118.1 (5)	H14C—C14B—H14D	108.3
O2—C5—O1	121.8 (5)	C16B—C15B—C14B	110.1 (19)
O2—C5—C1	121.2 (6)	C16B—C15B—H15C	109.6
O1—C5—C1	117.0 (5)	C14B—C15B—H15C	109.6
C3—C6A—C7A	110.2 (9)	C16B—C15B—H15D	109.6
C3—C6A—H6A	109.6	C14B—C15B—H15D	109.6
C7A—C6A—H6A	109.6	H15C—C15B—H15D	108.1
C3—C6A—H6B	109.6	C15B—C16B—H16D	109.5
C7A—C6A—H6B	109.6	C15B—C16B—H16E	109.5
H6A—C6A—H6B	108.1	H16D—C16B—H16E	109.5
C8A—C7A—C6A	109.4 (10)	C15B—C16B—H16F	109.5
C8A—C7A—H7A	109.8	H16D—C16B—H16F	109.5
C6A—C7A—H7A	109.8	H16E—C16B—H16F	109.5
C8A—C7A—H7B	109.8	Zn1—O1W—H1W	125.4
C6A—C7A—H7B	109.8	Zn1—O1W—H2W	121.6
H7A—C7A—H7B	108.2	H1W—O1W—H2W	112.2
C3—C6B—C7B	104 (3)	Zn1—O2W—H3W	129.7
C3—C6B—H6C	110.9	Zn1—O2W—H4W	116.4
C7B—C6B—H6C	110.9	H3W—O2W—H4W	113.1
C3—C6B—H6D	110.9	H5W—O3W—H6W	111.6
C7B—C6B—H6D	110.9	H7W—O4W—H8W	111.8
H6C—C6B—H6D	109.0	H9W—O5W—H10W	110.3
C8B—C7B—C6B	115 (3)	H11W—O6W—H12W	111.4
C8B—C7B—H7C	108.4		

### Hydrogen-bond geometry (Å, °)

**Table:**

*D*—H···*A*	*D*—H	H···*A*	*D*···*A*	*D*—H···*A*
O1—H1···O4	0.82	1.68	2.502 (6)	180
O7—H7···O6	0.82	1.63	2.445 (6)	176
N2—H2···O4W	0.86	1.88	2.737 (6)	171
N4—H4···O6W	0.86	2.03	2.863 (6)	162
O1W—H1W···O5W^i^	0.84	1.86	2.675 (6)	164
O1W—H2W···O8^ii^	0.84	1.89	2.720 (5)	170
O2W—H3W···O8^iii^	0.83	2.13	2.910 (6)	155
O2W—H4W···O2^iv^	0.83	2.00	2.802 (6)	161
O3W—H5W···O3^iv^	0.84	1.98	2.795 (8)	162
O3W—H6W···O3^v^	0.84	1.92	2.743 (8)	165
O4W—H7W···O3W	0.84	1.81	2.633 (9)	163
O4W—H8W···O7^vi^	0.84	2.16	2.877 (6)	143
O5W—H9W···O5^vi^	0.85	2.14	2.865 (6)	144
O5W—H10W···O4^vii^	0.85	1.98	2.812 (6)	169
O6W—H11W···O3W^vii^	0.84	1.74	2.574 (10)	174
O6W—H12W···O5W^viii^	0.84	2.27	2.789 (7)	120

Symmetry codes: (i) *x*+1, *y*, *z*−1; (ii) −*x*+1, −*y*+1, −*z*; (iii) *x*+1, *y*, *z*; (iv) −*x*+2, −*y*, −*z*+1; (v) *x*, *y*, *z*+1; (vi) −*x*+1, −*y*+1, −*z*+1; (vii) −*x*+1, −*y*, −*z*+1; (viii) *x*, *y*, *z*−1.
